# Ginsenoside Rb1-Enriched Saponin Fraction Inhibits M1 Macrophage Polarization by Suppression of TLR4 Trafficking in Metabolic Dysfunction-Associated Alcoholic Liver Disease

**DOI:** 10.3390/nu18142294

**Published:** 2026-07-13

**Authors:** Tae-Un Kim, Jae-Hyuk Yim, Woo Jun Kim, Seoung-Woo Lee, Hee-Yeon Kim, Kyung-Ku Kang, Min-Soo Seo, Man Hee Rhee, Su-Min Baek, Seong-Kyoon Choi, Jin-Kyu Park

**Affiliations:** 1 Department of Veterinary Pathology, College of Veterinary Medicine, Kyungpook National University, Daegu 41566, Republic of Korea; krytne1222@naver.com (T.-U.K.);; 2 Core Protein Resources Center, Daegu-Gyeongbuk Institute of Science and Technology (DGIST), Daegu 42988, Republic of Korea; 3 Preclinical Research Center, K-MEDI Hub, Daegu 41061, Republic of Korea; 4 College of Veterinary Medicine, Kyungpook National University, Daegu 41566, Republic of Korea; 5 Institute for Veterinary Biomedical Science, Kyungpook National University, Daegu 41566, Republic of Korea

**Keywords:** MetALD, red ginseng saponin fraction, TLR4 trafficking, competitive inhibitor

## Abstract

**Background/Objectives:** Metabolic dysfunction-associated alcoholic liver disease (MetALD) is a serious worldwide health concern, exhibiting metabolic dysfunction-associated lipid accumulation, alcohol-associated oxidative damage, and endotoxin-induced inflammation. Rb1-enriched red ginseng saponin fraction (RGSF) has been known to exhibit anti-inflammatory and anti-oxidative properties, but its role in MetALD remains to be fully elucidated. This study aims to investigate the specific mechanism of RGSF in the MetALD mouse model. **Methods:** The MetALD mouse model was administered with or without Rb1-RGSF for 7 weeks. Histopathological and molecular analyses, along with primary cell isolation, were conducted for in vivo and ex vivo investigations. M1 macrophage polarization was assessed by analyzing pro-inflammatory cytokine expression. NF-kB/p65 and TLR4 protein expression were measured before being visualized using immunofluorescence assays and confocal microscopy. **Results:** Histopathological examination revealed that RGSF treatment markedly reduced hepatic steatosis and attenuated inflammatory lesions in MetALD independent of oxidative stress. Notably, RGSF administration suppressed the LPS-induced internalization of surface TLR4. During the early inflammatory phase, RGSF prevented the LPS-mediated loss of the 130 kDa TLR4 form at the cell membrane, thereby limiting the generation of its 110 kDa cytoplasmic form. LPS-binding assay confirmed the direct interactions between TLR4 and RGSF. **Conclusions:** Collectively, these findings demonstrate that RGSF regulates TLR4 expression and trafficking, leading to the suppression of M1 macrophage polarization by inhibiting LPS–TLR4 surface interactions, thus exhibiting hepatoprotective effects.

## 1. Introduction

Steatotic liver disease encompasses fatty liver disease with various etiologies of steatosis, and it is classified based on its cause and risk factors. It includes metabolic dysfunction-associated steatotic liver disease (MASLD), metabolic dysfunction-associated alcoholic liver disease (MetALD), and alcohol-associated liver disease [1,2,3]. MetALD refers to patients with MASLD who consume at least 140 g/week and 210 g/week of alcohol for females and males, respectively [1,2,3]. Its pathogenesis involves liver steatosis and excessive hepatitis due to lipid metabolism dysfunction and alcohol consumption [1,2,4]. Despite the heightened risk of severe liver disorders, therapeutics for the complete recovery of chronic liver disease remain to be established. In MetALD, liver injury results from metabolic dysfunction-associated lipid peroxidation, alcohol-associated oxidative damage, and endotoxin-induced inflammation, making macrophage plasticity crucial to its pathogenesis [4]. Oxidative stress-induced hepatocyte degeneration releases diverse damage-associated molecular patterns (DAMPs), triggering the production of pro-inflammatory enzymes and cytokines, such as tumor necrosis factor-alpha (TNF-α), inducible nitric oxide synthase (iNOS), and interleukin-1β (IL-1β), promoting M1 macrophage differentiation [4,5].

Upon exposure to pro-inflammatory stimuli, NF-κB signaling is activated through site-specific phosphorylation and subsequent degradation of IκB by the IκB kinase (IKK) complex [6,7]. The NF-κB pathway is triggered by toll-like receptor 4 (TLR4) dimerization [7,8,9]. TLR4 has nine sites of N-linked glycosylation, which are crucial for cell surface expression of TLR4 and LPS binding [10]. Cellular TLR4 is present in two different isoforms: heavily glycosylated 130 kDa TLR4 present on the cell surface and partially glycosylated 110 kDa TLR4 found predominantly in the Golgi [11,12].

*Panax ginseng*, a traditional therapeutic herb in Eastern Asia, has been widely studied for its anti-inflammatory and antioxidant properties, particularly red ginseng derived from the *P. ginseng* root [13,14,15,16,17,18]. Ginsenosides, the primary bioactive components of red ginseng, are steroidal glycosides derived from triterpene dammarane, which contains 30 carbon atoms [18,19,20]. Ginsenosides are classified into subtypes (e.g., Rg1, Rg2, Rg3, Rb1, Rb2, Rc, Rd, Re, Rf, and Rh) based on carbohydrate side chains, as determined by high-performance liquid chromatography (HPLC) [19,21]. Previous studies highlight the therapeutic potential of the Rb1-enriched red ginseng saponin fraction (RGSF) in various inflammatory disorders [21,22,23,24,25,26]. However, its role in the pathogenesis of MetALD remains unclear. Therefore, this study aims to investigate the role of RGSF in TLR4 activation and subsequent macrophage polarization in hepatic inflammation associated with MetALD.

## 2. Material and Methods

### 2.1. Sample Preparation

RGSF from red ginseng extract was provided by Korea Ginseng Corporation (Daejeon, Republic of Korea). The stem (25%) and root (75%) of Korean Red Ginseng were extracted with distilled water and 55% ethanol (EtOH). The crude extract was concentrated by multiple extractions and dried in a vacuum drier. Its constituents were analyzed using HPLC (Supplementary Data S1). RGSF contained Rb1 (C_54_H_92_O_23_, 108.15 mg/g), Rc (C_53_H_90_O_22_, 44.84 mg/g), Rb2 (C_53_H_90_O_22_, 40.6 mg/g), Rg3s (C_42_H_72_O_13_, 34.14 mg/g), Re (C_48_H_82_O_18_, 29.6 mg/g), Rg1 (C_42_H_72_O_14_, 24.72 mg/g), Rg2s (C_42_H_72_O_13_, 23.07 mg/g), Rf (C_42_H_72_O_14_, 21.42 mg/g), Rh1 (C_36_H_62_O_9_, 21.42 mg/g), Rd (C_48_H_82_O_18_, 20.08 mg/g), and Rg3r (C_53_H_90_O_22_, 14.61 mg/g), totaling 382.65 mg/g (Supplementary Data S1).

### 2.2. Animal Experimental Design

Nine-week-old wild-type (WT) C57BL/6 male mice (Orient Bio Inc., Seongnam, Republic of Korea were used in this study. The mice were housed under controlled conditions at 22 °C ± 3 °C with a relative humidity of 50 ± 10%, and they were provided a maintenance diet and water ad libitum. MetALD was induced in mice as previously described by Babuta et al., 2024 [4]. The animals were randomly assigned to six experimental groups: (1) control diet (CD) + vehicle (*n* = 3); (2) CD + RGSF 250 mg/kg (*n* = 3); (3) CD + RGSF 500 mg/kg (*n* = 3); (4) Western diet + EtOH (WE) + vehicle (*n* = 5); (5) WE + RGSF 250 mg/kg (*n* = 5); and (6) WE + RGSF 500 mg/kg (*n* = 5). CD groups were administered a standard chow diet (20.8% protein, 67.7% carbohydrate, 11.5% fat), while WE groups were administered a western diet (17% protein, 43% carbohydrate, 41% fat) supplemented with EtOH in increasing concentrations (10% EtOH for the first week and 20% for the next 6 weeks) mixed with a sugar water solution (23.1 g/L fructose + 18.9 g/L glucose in 1 L of filtered tap water). RGSF was administered by oral gavage at 250 mg/kg or 500 mg/kg body weight five times per week for 7 weeks. Body weight and food/water intake were monitored throughout the study. After 7 weeks, all mice were euthanized, and liver tissue was collected for analysis. All animal experiments were conducted in accordance with the National Institutes of Health Guide for the Care and Use of Laboratory Animals. The study was approved by the Institutional Animal Care and Use Committee (IACUC) of Kyungpook National University (Approval No. 2022-0004, Approval Date 22 August 2022).

### 2.3. Histopathological Analysis

Liver tissue samples were fixed in 10% neutral buffered formalin, processed, and embedded in paraffin wax for histopathological analysis. Paraffin blocks were sectioned into 4 μm slices and stained with hematoxylin and eosin for microscopic investigation. Tissues fixed in 4% paraformaldehyde were rinsed with phosphate-buffered saline (PBS) and immersed in 30% sucrose in PBS for 3 days. The samples were then embedded in an optimal cutting temperature compound and sectioned into 3–10 μm thick slices for Oil-Red-O staining and immunofluorescence. Histopathological grading was conducted using the modified NASH Clinical Research Network (CRN) grading system (Supplementary Data S2).

### 2.4. Oil-Red-O Staining

Cryo-slides of frozen liver tissue were washed with PBS and incubated with filtered Oil-Red-O working solution for 20 min at RT. The slides were counterstained with 10% hematoxylin and covered with glycerin. Oil-Red-O positive areas were quantified with the ImageJ software (version 1.53k; National Institutes of Health, Bethesda, MD, USA). At least five random ×200-magnification fields were analyzed from each sample for grading and quantification.

### 2.5. Immunohistochemistry

Paraffin-embedded samples were sectioned into 3 μm thick slices for immunohistochemical analysis. Antigen retrieval was conducted using 3% hydrogen peroxide in methanol for 30 min at room temperature, followed by steaming in a citric acid buffer for an additional 30 min. After cooling, the slides were rinsed with distilled water and PBS for 3 min each, then incubated in a blocking solution (HRP-060, Zytomed Systems, GmbH, Berlin, Germany). The slides were incubated overnight with an anti-myeloperoxidase (MPO) primary antibody (rabbit polyclonal, 1:100, PA5-16672, Thermo Fisher Scientific, Waltham, MA, USA). After PBS washing, they were incubated with a broad-spectrum secondary antibody and a streptavidin-horseradish peroxidase conjugate (HRP-060, Zytomed Systems) for 10 min each. Visualization was conducted using diaminobenzidine (Vector Laboratories, Burlingame, CA, USA), followed by counterstaining with 10% hematoxylin. Negative controls were incubated with 1× PBS instead of the primary antibody to rule out nonspecific binding.

### 2.6. Immunofluorescence Staining and Confocal Microscopy

The frozen blocks were sectioned into 4 μm thick slices for immunofluorescence staining and confocal microscopy. After rinsing with PBS, the slides were permeabilized with 0.1% Triton X-100 in PBS, blocked with 5% donkey serum, and incubated with the primary antibody. The primary antibodies included anti-CD68 (rat monoclonal, 1:200, MCA1957GA, Bio-Rad, Hercules, CA, USA), iNOS (mouse monoclonal, 1:200, sc-7271, Santa Cruz Biotechnology, Dallas, TX, USA), p65 (rabbit monoclonal, 1:200, #8242, Cell Signaling Technology, Danvers, MA, USA), TLR4 (rabbit monoclonal, 1:200, D8L5W, Cell Signaling Technology, Danvers, MA, USA), and Alexa Fluor™ 555 phalloidin (a34055, Invitrogen, Carlsbad, CA, USA), all diluted in 5% donkey serum. The secondary antibodies included Alexa Fluor™ 488 donkey anti-rat IgG (ab150153, Abcam, Cambridge, UK), Alexa Fluor™ 488 donkey anti-rabbit IgG (a-21206, Invitrogen, Carlsbad, CA, USA), Alexa Fluor™ 555 donkey anti-mouse IgG (ab150106, Abcam, Cambridge, UK), and Alexa Fluor™ 555 donkey anti-rabbit IgG (ab150154, Abcam, Cambridge, UK). The slides were then mounted with ProLong Gold Antifade Reagent containing DAPI (Cell Signaling Technology, Danvers, MA, USA) for nuclear staining. Negative slides were prepared by omitting the primary antibody to assess nonspecific binding. Confocal microscopy was conducted using a Carl Zeiss LSM800 (Carl Zeiss AG, Oberkochen, Germany) super-resolution confocal laser-scanning microscope.

### 2.7. Triglyceride Assay

Frozen liver tissues were incubated overnight in isopropanol (20 μL/mg) at 4 °C. The samples were then centrifuged at 10,000 rpm for 15 min, and the supernatant was collected. Triglyceride levels were measured using an L-type triglyceride kit (Wako, Osaka, Japan), following the instructions of the manufacturers.

### 2.8. Lipid Peroxidation Measurement (TBARS)

Malondialdehyde (MDA) concentration as a product of lipid peroxidation was measured using a commercially available TBARS assay (Cayman Chemical, Ann Arbor, MI, USA). Using an MDA standard curve, concentrations in plasma samples were calculated. The TBARS assay was performed by combining the sample, sodium dodecyl sulfate (SDS) and a coloring reagent, which is intensified by the amount of MDA present in the sample. The samples were read using a fluorometer (530 nm excitation, 550 nm emission). Protein concentrations were determined using the DC Protein Assay Kit (Bio-Rad), and MDA levels were normalized to the protein concentration of each sample.

### 2.9. Western Blot Analysis

The protein concentration of cell lysates was measured using the DC Protein Assay Kit (Bio-Rad). Protein samples were separated by electrophoresis on a 10% sodium dodecyl sulfate-polyacrylamide gel and transferred to an Immobilon-P membrane (EMD Millipore Corporation, Billerica, MA, USA) using a wet transfer system. The membrane was blocked with 5% skim milk in Tris-buffered saline containing 0.1% Tween^®^ 20 (TBS-T) for 1 h at room temperature to eliminate nonspecific binding. It was then incubated overnight at 4 °C with primary antibodies, including anti-iNOS (mouse monoclonal, 1:200, sc-7271, Santa Cruz Biotechnology, Dallas, TX, USA), phospho-NF-κB p65 (rabbit polyclonal, 1:500, #3039s, Cell Signaling Technology, Danvers, MA, USA), NF-κB p65 (rabbit monoclonal, 1:500, #8242s, Cell Signaling Technology, Danvers, MA, USA), IκBα (rabbit monoclonal, 1:500, ab32518; Abcam, Cambridge, UK), TLR4 (rabbit monoclonal, 1:500, #14358s, Cell Signaling Technology, Danvers, MA, USA), and β-actin (mouse monoclonal, 1:2000, sc-47778, Santa Cruz Biotechnology, CA, USA). The membrane was then incubated with secondary antibodies, goat anti-rabbit (401393, Calbiochem, San Diego, CA, USA) and goat anti-mouse (401253, Calbiochem), diluted in 5% skim milk. Bands were detected using an enhanced chemiluminescence (ECL) solution (Translab, Seoul, Korea) and visualized with the FUSION SOLO S (Vilber, Eberhardzell, Germany).

### 2.10. Quantitative Reverse Transcription-Polymerase Chain Reaction

The isolated liver tissue and cellular RNA were converted into cDNA using the RT Prime kit (EBT-1520, ELPIS Biotech, Daejeon, Republic of Korea). Gene expression levels were quantified using qRT-PCR, with 18S rRNA as the loading control. Supplementary Data S3 depicts the primer sequences used in the study.

### 2.11. In Vitro Experiment

Murine macrophage RAW 264.7 cells were obtained from the Korean Cell Line Bank (KCLB, Seoul, Republic of Korea; KCLB No. 40071). Cells were seeded in a 12-well plate at a density of 1 × 10^5^ cells/well. After 24 h, the cells were treated with 25 or 50 μg/mL RGSF diluted in DPBS, with or without 1 μg/mL LPS (L2880; Sigma-Aldrich, St. Louis, MO, USA) before cell harvest. Cells were then harvested for gene and protein extraction and immunofluorescence staining.

### 2.12. Cell Viability Assay

Viability of LPS- and RGSF-treated cells was measured using Cell Counting Kit-8 (CCK-8; CK04, Dojindo Laboratories, Kumamoto, Japan). Briefly, cells were seeded in a 96-well culture plate (6250 cells per well) and incubated at 37 °C for 24 h, and the media was replaced with C-DMEM with gradual concentrations of RGSF w/o LPS at 37 °C for 6 h. CCK-8 solution (10 μL per well) was added, followed by an additional incubation for 3 h. The absorbance was measured at 450 nm.

### 2.13. Inhibition of LPS Binding to TLR4

RAW 264.7 cells were incubated with RGSF at 25 and 50 μg/mL for 1 h and then stimulated with Alexa Fluor 488-LPS (L23351, Invitrogen, Carlsbad, CA, USA) at 10 μg/mL for 20 min. Cells were then fixed with 4% paraformaldehyde. After rinsing with PBS, the cells were permeabilized with 0.1% Triton X-100 in PBS, blocked with 5% donkey serum, and incubated with TLR4 primary antibody (rabbit monoclonal, 1:200, D8L5W, Cell Signaling Technology, Danvers, MA, USA). Alexa Fluor™ 555 donkey anti-rabbit IgG (1:500, ab150154, Abcam, Cambridge, UK) was used as secondary antibody.

### 2.14. Primary Kupffer Cell Isolation

Primary Kupffer cells (KCs) were isolated from the livers of 10-week-old male C57BL/6 mice. Kupffer cell isolation was performed using the collagenase liver perfusion system as described in the previous study [20,27]. Liver perfusion was conducted using 1× EGTA buffer and collagenase type II (LS004176; Worthington Biochemical Corporation, Lakewood, NJ, USA) to obtain liver cell suspensions. Perfusion time and flow rate were adjusted based on cell type. Non-parenchymal cells (NPCs) were isolated from hepatocytes by centrifugation at 50× *g* for 5 min. The supernatant was further centrifuged at 350× *g* for 10 min to collect NPC pellets, which were resuspended in Hank’s balanced salt solution (HBSS, LB003-01; Welgene, Gyeongsan, Republic of Korea). The suspension underwent density gradient centrifugation using 25% and 50% Percoll^TM^ (17-0891-02; Cytiva, Uppsala, Sweden) diluted in DPBS. KCs were seeded in 12-well plates with RPMI-1640 medium supplemented with 10% Fetal bovine serum (FBS; 16000044, Gibco, Thermo Fisher Scientific, Waltham, MA, USA) and 1% streptomycin/penicillin/amphotericin B (LS-203-01; WELGENE Inc., Gyeongsan, Republic of Korea). After 30 min, non-adherent cells were removed by gently washing with DPBS. After 24 h, isolated KCs were treated with 25 or 50 μg/mL RGSF, with or without 1 μg/mL LPS. Cells were washed with DPBS and harvested 24 h post-treatment for immunofluorescence staining and Western blot analysis.

### 2.15. Statistical Analysis

All data in this study were presented as the mean ± standard deviation (SD). Statistical analysis was conducted using the unpaired Student’s *t*-test and one-way ANOVA followed by post hoc testing. Statistical significance was evaluated with GraphPad Prism 5.0 (GraphPad Software Inc., San Diego, CA, USA) and GraphPad InStat 3.0 (GraphPad Software Inc.). NS: not significant (*p* > 0.05); * (*p* < 0.05); and ** (*p* < 0.01).

## 3. Results

### 3.1. RGSF Alleviated MetALD-Associated Lesions Dose-Dependently in MetALD Mouse Model

**Figure 1:**
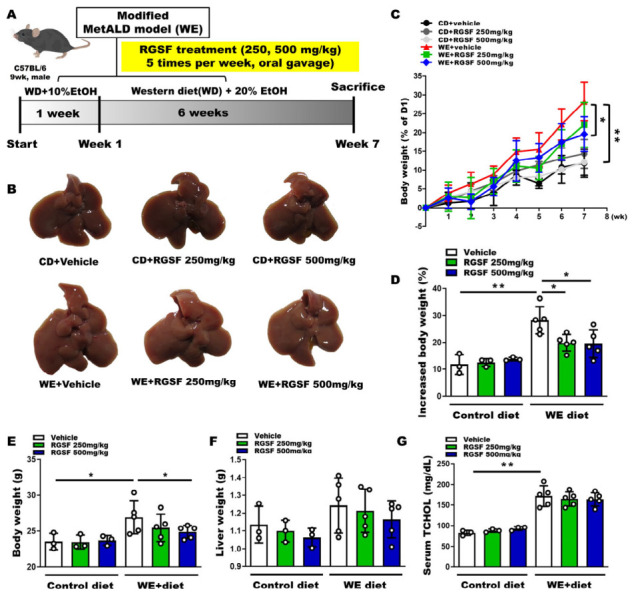
RGSF treatment reduced body and liver weight in MetALD liver: (**A**) Schematic of the animal experiment. C57BL/6 mice were fed a Western diet with ethanol-supplemented high-sugar water *ad libitum*, with or without oral RGSF administration. (**B**) Macroscopic liver images. (**C**) Body weight changes from day 1 (%). (**D**) Increased body weight (% of day 1). (**E**) The body weight on the last day (g). (**F**) The liver weight (g). (**G**) The serum levels of total cholesterol. Data are presented as mean ± standard deviation (* *p* < 0.05, ** *p* < 0.01). MetALD: Metabolic dysfunction-associated Alcoholic Liver Disease; RGSF: red ginseng saponin fraction; EtOH: ethanol; CD: Control diet; WE: Western diet + EtOH; TCHOL: total cholesterol.

Throughout the experimental period (Figure 1A), the WE + vehicle group exhibited significantly higher body weight than that of the CD + vehicle group (Figure 1C). RGSF treatment reduced body weight in the WE + RGSF groups (Figure 1C,E). Similarly, the percentage changes in body weight were reduced in the WE + RGSF groups compared with the WE + vehicle group (Figure 1D). Gross liver observation showed that the WE + vehicle group had enlarged pale livers, indicating steatotic liver injury (Figure 1B). Although the difference did not reach statistical significance, liver weight was higher in the WE group than in the CD group (Figure 1F). In contrast, RGSF administration led to a reduction in liver weight compared to that of the WE + vehicle group (Figure 1F). Serum total cholesterol levels followed a similar trend, increasing in the WE + vehicle group and decreasing in the WE + RGSF groups, although the difference did not reach statistical significance (Figure 1G).

### 3.2. MetALD-Associated Hepatic Lesions Were Alleviated by RGSF Treatment

**Figure 2:**
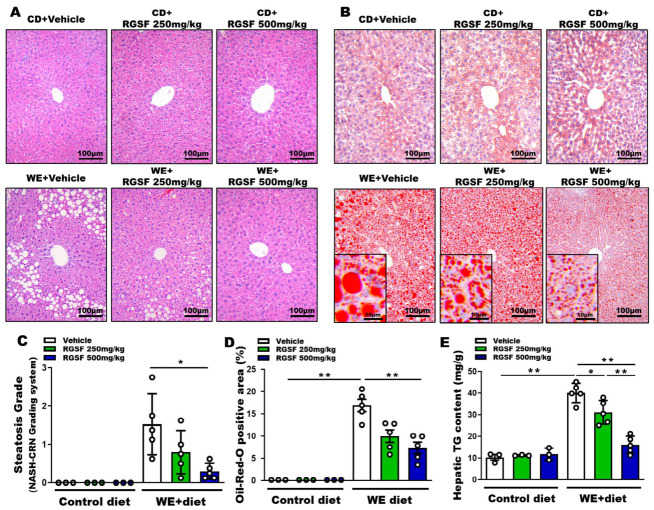
RGSF treatment reduced hepatic steatosis: (**A**) Representative H&E images from each mouse group. Scale bar = 100 μm. (**B**) Oil Red O-stained liver sections. Scale bar = 100 μm. (**C**) NASH-CRN scoring of liver steatosis. (**D**) Quantification of Oil Red-O-positive areas. (**E**) Hepatic TG contents across all groups. Data are presented as mean ± standard deviation (* *p* < 0.05, ** *p* < 0.01). NASH-CRN: nonalcoholic steatohepatitis clinical research network; TG: triglyceride.

In histopathological analysis, the WE + vehicle group showed aggravated hepatic steatosis, characterized by lipid droplet accumulation, compared to that of the CD + vehicle group (Figure 2A). In contrast, the WE + RGSF groups showed a reduction in lipid accumulation (Figure 2A). A similar trend was observed in the histopathological grading of the liver tissue, where the WE + vehicle group showed a significantly higher hepatic steatosis grade than that of the CD + vehicle group, which was significantly alleviated by RGSF treatment (Figure 2C). Oil Red O staining further confirmed this effect, as WE + RGSF groups showed a significant reduction in lipid droplet size and number compared to that in the WE + vehicle group (Figure 2B,D). Consistent with the histopathological analysis, the WE + vehicle group showed significantly elevated hepatic TG levels compared to those of the CD + vehicle group, while RGSF treatment significantly reduced hepatic TG levels in a dose-dependent manner (Figure 2E).

### 3.3. RGSF Treatment Alleviated MetALD Liver by Suppressing M1 Macrophage Polarization Rather than Oxidative Stress

**Figure 3:**
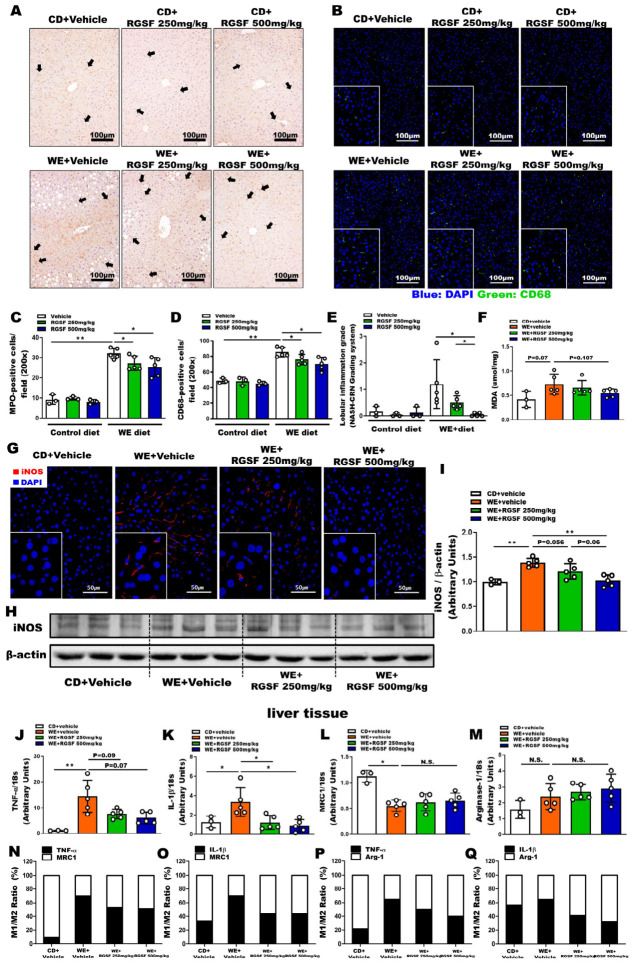
RGSF treatment significantly reduced infiltration of MPO- and CD68-positive cells independent of oxidative stress in MetALD liver: (**A**) Immunohistochemistry for MPO. MPO-positive cells are indicated by arrows. Scale bar = 100 μm. (**B**) Immunofluorescence staining for CD68 (green) and nuclei (DAPI, blue). Scale bar = 100 μm. (**C**) NASH-CRN scoring for hepatic lobular inflammation. (**D**) MPO-positive cells per 200× magnification field. (**E**) CD68-positive cells per 200× magnification field. (**F**–**H**) iNOS expression in liver tissue. (**F**) MDA concentration of the liver tissue. (**G**) Immunofluorescence staining of iNOS. (**H**) Protein expression of iNOS. (**I**) Quantification data of iNOS expression. (**J**,**K**) M1 macrophage marker gene expression in liver tissue. (**J**) TNF-α and (**K**) IL-1β mRNA levels. (**L**,**M**) M2 macrophage marker gene expression in liver tissue. (**L**) MRC1 and (**M**) Arg-1 mRNA levels. (**N**–**Q**) M1/M2 ratio based on mRNA levels. (**N**) TNF-α/MRC1 and (**O**) IL-1β/MRC1 expression ratios. (**P**) TNF-α/ Arg-1 and (**Q**) IL-1β/ Arg-1 expression ratios. Data are presented as mean ± standard deviation (* *p* < 0.05, ** *p* < 0.01). MPO: myeloperoxidase; CD68: cluster of differentiation 68; iNOS: inducible nitric oxide synthase; TNF-α: tumor necrosis factor alpha; IL-1β: interleukin 1 beta; MRC1: mannose receptor C-type 1; Arg-1: arginase 1.

We then investigated inflammatory cell infiltrations histopathologically in all mouse groups (Figure 3A–E). Histopathological inflammatory grading was significantly increased in the WE group compared with the CD group, whereas RGSF treatment significantly reduced the inflammatory grade, indicating attenuation of WE-induced hepatic inflammation (Figure 3E). The number of MPO- and CD68-positive cells was significantly higher in the liver parenchyma of the WE + vehicle group (Figure 3C,D). However, RGSF treatment significantly reduced MPO- and CD68-positive cells (Figure 3A,D). In CD groups, RGSF administration did not induce additional inflammatory infiltration beyond residual KCs (Figure 3D), indicating no toxic effects on the liver. To examine hepatocellular injury and inflammatory activation from oxidative stress, the MDA concentration of liver tissue was examined (Figure 3F). WE + vehicle group showed a higher MDA concentration compared to that in the CD + vehicle group, which showed a tendency to decrease in the WE + RGSF groups (Figure 3F). However, no significant change was detected, which led to further examination of inflammatory pathways, especially macrophage polarization. iNOS expression, a marker of M1 macrophage polarization, was detected in liver tissue (Figure 3G–I). Immunofluorescence analysis showed increased iNOS expression in the WE + vehicle group compared to that in the CD + vehicle group, which decreased following RGSF treatment (Figure 3G). Consistent with the immunofluorescence assay, the WE + vehicle group exhibited the highest iNOS protein expression among all groups (1.39-fold change relative to the CD + vehicle group, *p*-value = 0.0005; Figure 3H,I). In contrast, RGSF-treated groups showed significantly lower iNOS expression than the WE + vehicle group (−1.35-fold change in the WE + RGSF 500 mg/kg group relative to the WE + vehicle group, *p*-value = 0.005; Figure 3H,I). MetALD significantly increased mRNA expression of M1 macrophage markers, TNF-α (Figure 3J) and IL-1β (Figure 3K), which decreased following RGSF treatment. However, M2 macrophage markers, MRC1 (Figure 3L) and arginase-1 (Figure 3M), remained unaffected by RGSF administration. The M1/M2 macrophage polarization ratio in the liver tissue was measured by evaluating the ratio of TNF-α and IL-1β expression to MRC1 (Figure 3N,O) and arginase-1 (Figure 3P,Q). The WE + vehicle group showed significantly elevated M1/M2 ratios compared to those of the CD + vehicle group. However, WE + RGSF groups showed reduced M1/M2 ratios compared to those of the WE + vehicle group (Figure 3N–Q).

### 3.4. RGSF Suppressed M1 Polarization of RAW 264.7 Cells and Primary Isolated Kupffer Cells

**Figure 4:**
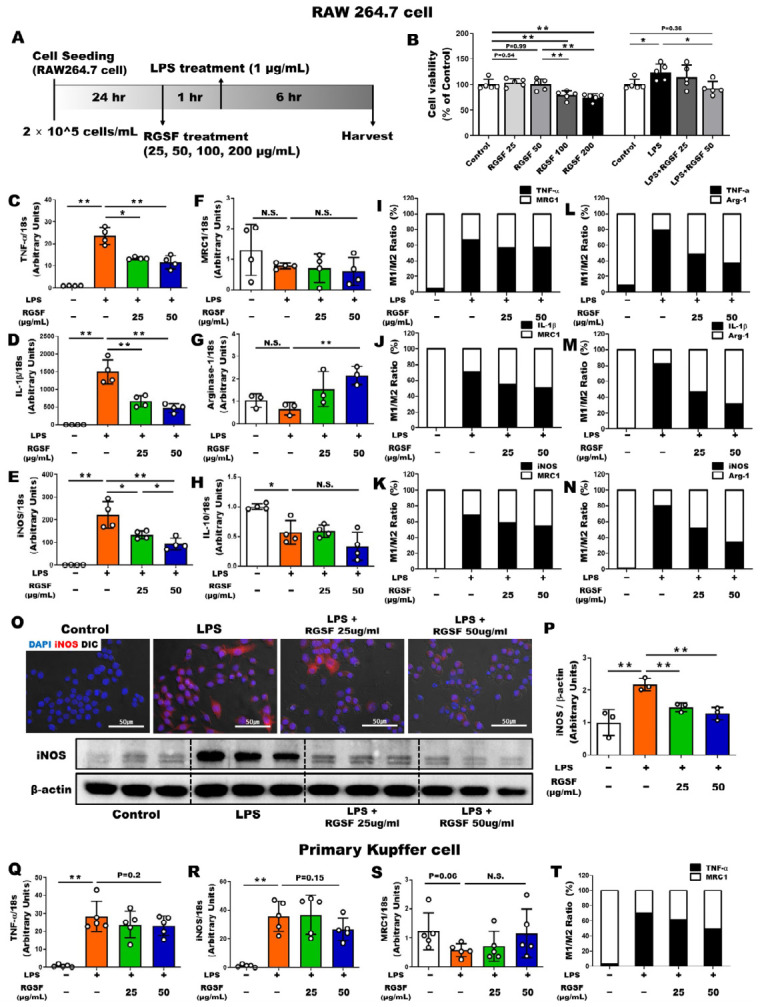
RGSF treatment inhibited LPS-induced M1 polarization without affecting M2 polarization: (**A**) Experimental scheme for LPS and RGSF treatment on RAW 264.7 cells. (**B**) Cell viability test for LPS and RGSF treatment. (**C**–**E**) Gene expression of M1 macrophage markers in LPS-treated murine macrophages. (**C**) TNF-α, (**D**) IL-1β, and (**E**) iNOS mRNA levels. (**F**–**H**) Gene expression of M2 macrophage markers. (**F**) MRC1, (**G**) IL-10, and (**H**) Arg-1 mRNA levels. (**I**–**N**) M1/M2 ratio based on mRNA levels. (**I**) TNF-α/MRC1, (**J**) IL-1β/MRC1, (**K**) iNOS/MRC1, (**L**) TNF-α/ Arg-1, (**M**) IL-1β/ Arg-1, and (**N**) iNOS/Arg-1 expression ratios. (**O**) Immunofluorescence and protein expression of iNOS. (**P**) Quantification data of iNOS expression. (**Q**–**T**) Gene expression of macrophage polarization markers in primary Kupffer cells. (**Q**) TNF-α, (**R**) iNOS, and (**S**) MRC1 mRNA levels. (**T**) M1/M2 ratio in primary KCs following LPS and RGSF treatment. Data are presented as mean ± standard deviation (N.S. *not significant*, * *p* < 0.05, ** *p* < 0.01). LPS: lipopolysaccharide; IL-10: interleukin-10. Different bar colors represent the different experimental groups corresponding to the treatment conditions indicated below the graph.

The murine macrophage RAW 264.7 cell line was used to investigate the role of RGSF in macrophage polarization (Figure 4A). To determine the effect of LPS and RGSF on cell viability, a CCK-8 cell viability assay was performed (Figure 4B). The cytotoxic effect was tested to confirm appropriate concentration ranges, and RGSF-treated cells did not show significant changes in cell viability by RGSF concentration up to 50 μg/mL (Figure 4B). The non-toxic concentrations (25, 50 μg/mL) were used for experiments. When treated with LPS for 6 h, cell viability significantly increased in the LPS-treated group. RGSF (up to 50 μg/mL) attenuated the LPS-induced increase in cell viability, indicating suppression of LPS-induced macrophage activation without cytotoxicity (Figure 4B). qRT-PCR analysis showed that M1 macrophage markers, such as TNF-α, IL-1β, and iNOS, were significantly higher in the LPS-treated group than in the control group (Figure 4C–E). However, RGSF treatment reduced TNF-α, IL-1β, and iNOS mRNA levels (Figure 4C–E). In contrast, RGSF treatment had no remarkable effect on MRC1 and IL-10 mRNA levels, though Arg-1 showed a significant increase in the WE+RGSF 500 mg/kg group compared with the WE + vehicle group (Figure 4F–H). The M1/M2 macrophage polarization ratio was determined using TNF-α, IL-1β, and iNOS as M1 markers and MRC1 and arginase-1 as M2 markers (Figure 4I–N). Consistent with the in vivo experiment, the LPS-treated group showed a significantly higher M1/M2 polarization ratio than that of the control group. In contrast, RGSF-treated groups showed a reduction in the M1/M2 polarization ratio compared with those of the LPS-treated groups (Figure 4I–N). Immunofluorescence and Western blot analyses further confirm iNOS expression in LPS- and RGSF-treated cells. LPS-induced iNOS protein expression (2.17-fold increase relative to the control group, *p*-value ≤ 0.01) was reduced following RGSF treatment (1.69-fold decrease in the high RGSF group relative to the LPS group, *p*-value ≤ 0.01), a trend consistently observed in immunofluorescence staining of iNOS, indicating a regulatory role of RGSF in M1 polarization (Figure 4O,P). Gene expression analysis and M1/M2 polarization ratio measurements in primary KCs isolated from WT C57BL/6 mice showed results consistent with in vivo and in vitro experiments (Figure 4Q–T).

### 3.5. RGSF Suppressed LPS-Induced NF-κB Activation in RAW 264.7 Cells and Primary Kupffer Cells

**Figure 5:**
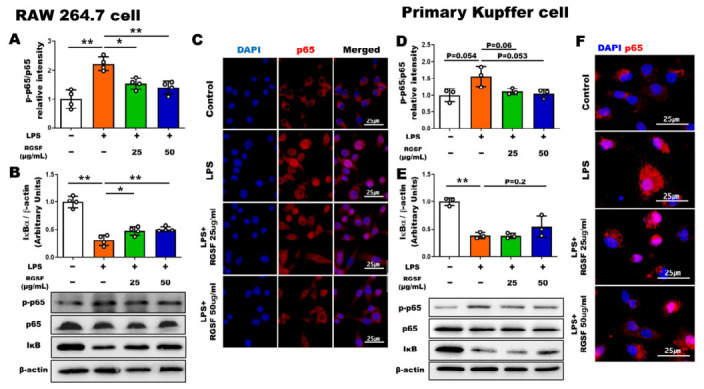
RGSF treatment downregulated LPS-induced NF-κB phosphorylation and IκB degradation: (**A**) NF-κB and (**B**) IκB expression levels in LPS- and RGSF-treated murine macrophages. (**C**) Immunofluorescence analysis of NF-κB. (**D**) NF-κB and (**E**) IκB expression levels in LPS- and RGSF-treated primary KCs. (**F**) Immunofluorescence analysis of NF-κB nuclear translocation in primary KCs. Data are presented as mean ± standard deviation (* *p* < 0.05, ** *p* < 0.01). p65: Nuclear factor kappa B subunit p65; p-p65: phosphorylated p65; IκB: inhibitor of kappa B. Different bar colors represent the different experimental groups corresponding to the treatment conditions indicated below the graph.

The NF-κB pathway, a key regulator of the pro-inflammatory cascade, was analyzed to investigate the regulatory roles of RGSF in M1 macrophage polarization. LPS stimulation increased total NF-κB/p65 phosphorylation (2.58-fold increase relative to the control group, *p*-value = 0.0001), which was suppressed by RGSF treatment (1.78-fold decrease in the high RGSF group relative to the LPS group, *p*-value = 0.0006; Figure 5A). Similarly, IκBα degradation by LPS stimulation (3.22-fold decrease relative to the control group, *p*-value = 0.001) was suppressed in RGSF-treated groups (1.64-fold increase in the high RGSF group relative to the LPS group, *p*-value = 0.008), consistent with the suppression of NF-κB phosphorylation (Figure 5B). Immunofluorescence staining of p65 showed that RGSF treatment reduced LPS-induced nuclear translocation of cytoplasmic p65 (Figure 5C). To further confirm these effects, primary KCs were isolated and analyzed (Figure 5D,E). Consistent with the results in RAW 264.7 cells, LPS-treated primary KCs showed a decrease in p65 phosphorylation (1.5-fold decrease in the high RGSF group relative to the LPS group, *p*-value = 0.053) and an increase in IκBα expression (1.41-fold increase in the high RGSF group relative to the LPS group, *p*-value = 0.233) following RGSF treatment (Figure 5D,E). Immunofluorescence staining of p65 further revealed that nuclear translocation of p65 was lower in RGSF-treated KCs than in LPS-treated KCs (Figure 5F).

### 3.6. RGSF Suppressed the Activation of TLR4 in RAW 264.7 Cells

**Figure 6:**
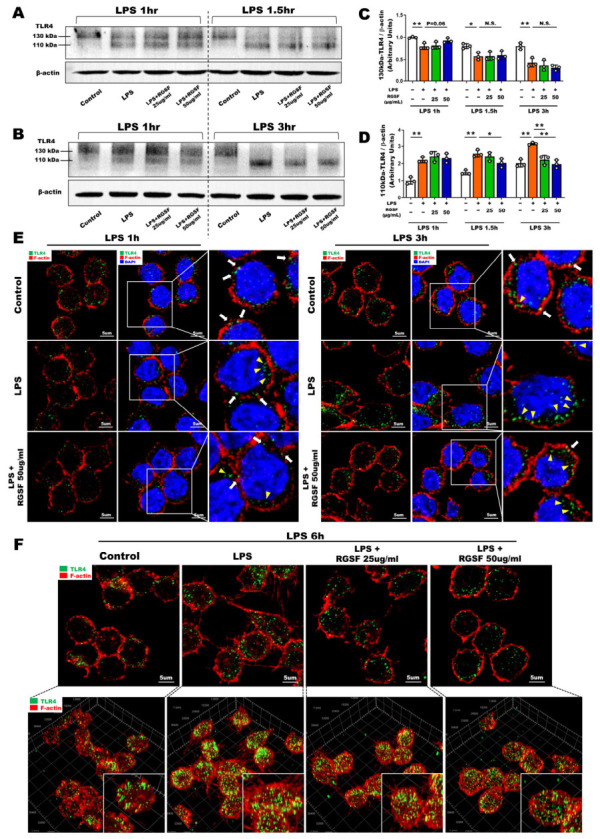
TLR4 expression levels following LPS and RGSF treatment: (**A**,**B**) TLR4 expressions of LPS-and RGSF-treated RAW 264.7 cells. (**C**) Protein expression of 130 kDa form of TLR4. (**D**) Protein expression of 110 kDa form of TLR4. (**E**) Confocal microscopy images of TLR4 in LPS- and RGSF-treated cells. Cell surface TLR4 (white arrow) and cytoplasmic TLR4 (yellow arrowhead). (**F**) Confocal microscopy images of cell surface and cytoplasmic TLR4 in RAW 264.7 cells with 6 h LPS stimulation w/o RGSF treatment. Data are presented as mean ± standard deviation (N.S. *not significant*, * *p* < 0.05, ** *p* < 0.01). TLR4: toll-like receptor 4. Different bar colors represent the different experimental groups corresponding to the treatment conditions indicated below the graph.

We investigated TLR4 activation to assess the effects of RGSF on the pro-inflammatory cascade. The initial and prolonged phases of inflammation were evaluated by examining TLR4 expression in LPS-treated cells. During the initial phase of LPS-induced inflammation, the 130 kDa N-glycosylated form of TLR4, located on the cell membrane, significantly decreased after LPS treatment (1.34-fold decrease vs. control, *p*-value = 0.008; Figure 6A,C). RGSF treatment showed a tendency to restore 130 kDa TLR4 expression compared with the LPS group, although this difference did not reach statistical significance (1.17-fold increase in high RGSF group relative to LPS group, *p*-value = 0.06; Figure 6C). Conversely, the 110 kDa cytoplasmic form of TLR4 increased after LPS stimulation (1.8-fold increase vs. control, *p*-value = 0.001; Figure 6D). RGSF partially attenuated this increase, with a significant reduction observed only at selected time points (Figure 6D). In the prolonged phase of LPS exposure, the 130 kDa TLR4 was depleted in LPS-treated groups but remained stable in control groups (1.34-fold decrease in the LPS group relative to the control group, *p*-value = 0.007; Figure 6B,C). Conversely, cytoplasmic TLR4 expression significantly increased in LPS-treated groups (1.63-fold increase relative to the control group, *p*-value = 0.001) and decreased dose-dependently following RGSF treatment (1.8-fold decrease in the high RGSF group relative to the LPS group, *p*-value = 0.001; Figure 6B,D). Confocal microscopy revealed reduced surface TLR4 levels during the initial phase up to 3 h of LPS treatment compared to those of the control groups. In contrast, surface TLR4 levels were maintained in the RGSF-treated group (Figure 6E). Cytoplasmic TLR4 levels increased in LPS-treated macrophages compared to those in the control group, while RGSF treatment showed lower levels of cytoplasmic TLR4 than that of the LPS-treated group (Figure 6E). Prolonged incubation with LPS and RGSF for 6 h significantly increased cytoplasmic TLR4 expression levels in the LPS-treated group compared to the control group, while RGSF treatment reduced cytoplasmic TLR4 expression dose-dependently (Figure 6F).

### 3.7. RGSF Suppressed LPS Binding to TLR4 in RAW 264.7 Cells

**Figure 7:**
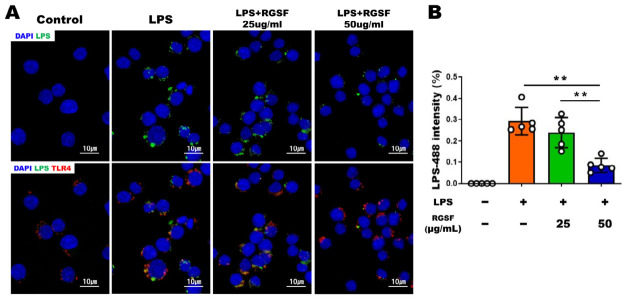
Suppressed LPS-TLR4 binding by RGSF treatment**:** (**A**) Immunofluorescence for the analysis of RGSF effect on LPS-TLR4 binding (LPS: green; TLR4: red). (**B**) Quantification data of cell-bound LPS. Data are presented as mean ± standard deviation (** *p* < 0.01).

As RGSF had suppressive effects on cell surface TLR4 depletion, we examined the binding of Alexa Fluor 488-conjugated LPS to RAW 264.7 cells with or without RGSF treatment (Figure 7A,B). As a result, RGSF-treated cells showed a significant decrease in LPS-TLR4 binding compared with the LPS group (Figure 7A). Intensity of cell-bound fluorescent LPS was also significantly decreased in cells treated with RGSF 50 μg/mL (3.45 fold-decrease in the high RGSF group relative to the LPS group, *p* = 0.0002, 2.82 fold-decrease in the high RGSF group relative to the low RGSF group, *p* = 0.002; Figure 7B), indicating that LPS-TLR4 binding was significantly suppressed in the RGSF-treated group (Figure 7A,B).

## 4. Discussion

MetALD contributes to the pathogenesis of both metabolic dysfunction and alcohol-induced inflammation. During MetALD development, hepatocytes undergo excessive oxidative stress from both ethanol metabolism and metabolic overload such as lipid peroxidation, ER stress, and mitochondrial dysfunction [28]. ROS generated by ethanol metabolism mediated by CYP2E1 and β-oxidation of excess fatty acids lead to hepatocyte disruption and DAMPs production [5,28,29]. Moreover, KCs are activated by DAMPs, leading to increased pro-inflammatory cytokine production and neutrophil and macrophage infiltration [5,28,29,30,31]. The pro-inflammatory cascade involves multiple pathways, including TLR4 and NF-κB activation, which promote M1 macrophage polarization and pro-inflammatory cytokine release, especially IL-6, TNF-α, IL-1β, and iNOS [5,8,32,33]. Released iNOS stimulates NO production, leading to a surge in •NO/ONOO− production, increasing reactive nitrogenous species (RNS) and ROS production and subsequent progression of the inflammatory cascade [33,34]. The therapeutic mechanism of RGSF on TLR4-mediated inflammation in MetALD mice was evaluated in this study.

In the animal experiment, RGSF alleviated steatosis and inflammatory lesions in MetALD mice. Reductions in liver and body weight, along with histopathological investigations showing lower steatosis and lobular inflammation grades in RGSF-treated groups, suggest a hepatoprotective effect against MetALD-induced liver damage. Immunohistochemical and immunofluorescence analyses of neutrophils and macrophages showed reduced macrophage recruitment in the livers of RGSF-treated MetALD groups. Since hepatic inflammation plays a critical role in the progression of hepatic steatosis and MetALD [35,36,37], these findings suggest that RGSF suppresses hepatic lipid accumulation in MetALD by attenuating hepatic inflammation. We assumed that the inflammatory response in MetALD could be associated with oxidative stress and conducted a TBARS assay to measure MDA concentrations. The MDA levels tended to increase in the WE + vehicle group and decrease in the WE + RGSF groups; however, the changes were not statistically significant. Consequently, we concluded that the concentration of RGSF used in our animal experiment had more effects on the regulation of the inflammatory pathway rather than the oxidative stress-related pathway. Although MDA is a widely used biomarker of oxidative stress in MetALD [38,39], RGSF treatment at 250 and 500 mg/kg produced little effect on MDA levels. Instead, the beneficial effects of RGSF were primarily associated with the suppression of M1 macrophage polarization. Additionally, the RGSF-treated CD groups exhibited no changes in inflammation or steatosis grades, indicating that RGSF alleviates MetALD-associated liver injury without toxicity. Several studies have reported that the pharmacokinetics of RGSF are rapidly absorbed, metabolized and eliminated [40,41]. The phenotypes observed in our study, combined with the pharmacokinetics of RGSF, highlight the safety of RGSF in MetALD.

Macrophages undergo polarization into distinct activation states depending on conditions, with two main phenotypes: the pro-inflammatory M1 and the anti-inflammatory M2 type [27,42,43]. KCs, the liver-residual macrophages, typically exhibit an M2 phenotype under non-inflammatory conditions [44,45]. However, under inflammatory stimuli, they shift toward the M1 phenotype, producing pro-inflammatory cytokines such as TNF-α, IL-1β, and iNOS [27,44]. The M1 polarization of KCs is induced by metabolic fluctuations in adipokines, free fatty acids, or gut-derived LPS [5,43,44,45]. M2-type KCs, induced by IL-4 or adiponectin, alleviate liver injury and promote M1 macrophage apoptosis, exhibiting anti-inflammatory properties [42]. Consistent with the findings of previous studies, MetALD groups showed increased gene expression of M1 macrophage markers, TNF-α and IL-1β. In this study, RGSF administration mitigated this increase in M1 markers. In contrast, M2 macrophage markers remained unchanged following RGSF treatment, confirming its downregulatory effect on pro-inflammatory cytokines in MetALD mice. Similarly, RGSF markedly downregulated LPS-induced transcriptional activation of pro-inflammatory cytokines in murine macrophages. RGSF also reduced LPS-induced iNOS upregulation at gene and protein levels. Macrophage-derived iNOS is crucial to the pro-inflammatory response, generating nitric oxide [46,47]. The dose-dependent reduction in iNOS expression in liver tissue and macrophages suggests that RGSF inhibits pro-inflammatory KC polarization into the M1 phenotype.

Further analysis focused on the NF-κB signaling pathway, as previous studies report that TLR4-mediated NF-κB and MAPK activation induces iNOS expression and modulates macrophage polarization into the pro-inflammatory M1 phenotype [46]. Upon LPS stimulation, IκB degradation and site-specific phosphorylation by the IKK complex facilitate nuclear translocation of p65/p50, leading to pro-inflammatory cytokine production [6,7]. In this study, RGSF markedly reduced p65 phosphorylation and IκB degradation in LPS-treated macrophages, thereby ameliorating pro-inflammatory macrophage polarization in primary KCs isolated from murine liver. The reduction in NF-κB signaling activation prompted an investigation into TLR4, a key receptor involved in NF-κB activation.

Previous studies reveal two forms of cellular TLR4 protein, one with a molecular weight of 130 kDa and the other with 110 kDa [10,11]. This difference results from N-linked glycosylation, which is essential for TLR4 surface trafficking. The 130 kDa TLR4, localized at the cell surface, exists in a heavily glycosylated form in the presence of MD-2, while the110 kDa form, found in the cytoplasm, remains partially glycosylated [10,11,12,48]. During the initial phase of inflammation, the 130 kDa TLR4 is stimulated by DAMPs or pathogen-associated molecular patterns, such as LPS, a key factor in the pathogenesis of alcohol-associated liver disease [8,9]. Upon LPS stimulation, TLR4 recruits adaptor molecules such as MyD88 and TRIF, leading to NF-κB activation and pro-inflammatory cytokine production [8,9]. The activation of NF-κB and MAPK signaling further enhances TLR4 transcription, creating a positive feedback loop that upregulates TLR4 expression. Concurrently, LPS-bound surface TLR4 undergoes endocytosis, binding to TRAM-TRIF and transitioning into a deglycosylated cytoplasmic form. Therefore, 130 kDa expression decreases during the initial phase of LPS stimulation due to endocytosis, while prolonged LPS exposure upregulates cytoplasmic TLR4 expression in macrophages. Previous studies have confirmed the anti-inflammatory effects of various ginsenosides through inhibition of the TLR4 signaling pathway [15,49,50,51]. In this study, RGSF treatment suppressed the decline in surface TLR4 expression during the early phase of LPS stimulation, indicating inhibition of LPS-induced TLR4 activation. Additionally, RGSF downregulated cytoplasmic TLR4 expression during prolonged LPS stimulation, suggesting its suppression of NF-κB activation following LPS exposure.

Here, RGSF inhibited LPS-induced TLR4 activation at the cell surface and suppressed intracellular TLR4 expression driven by inflammatory cytokines. We next hypothesized that RGSF would have an inhibitory effect on LPS-TLR4 binding. The LPS-binding assay confirmed the inhibitory effects of RGSF on LPS-TLR4 binding, suggesting that RGSF inhibits the direct binding of LPS and the TLR4-MD-2 complex on the cell surface. Moreover, IL-4-induced M2 polarization was suppressed by RGSF treatment (unpublished data), raising the possibility that RGSF could inhibit various macrophage surface receptors.

## 5. Conclusions

Collectively, RGSF significantly alleviates MetALD by downregulating M1 macrophage polarization through the suppression of the TLR4 signaling pathway and subsequent NF-κB activation. Its effects may extend beyond hepatic inflammation to systemic inflammation by suppressing surface TLR4 activation and prolonged cytoplasmic TLR4 expression in macrophages. These findings highlight the regulatory role of RGSF in TLR4 expression by suppressing LPS-induced TLR4 shift from the heavily glycosylated 130 kDa form into the partially glycosylated 110 kDa form in the inflammatory pathway of MetALD. Finally, we confirm that RGSF suppresses the binding of LPS and TLR4, supporting its potential as a therapeutic agent for MetALD.

## Data Availability

The original contributions presented in this study are included in the article/Supplementary Materials. Further inquiries can be directed to the corresponding author.

## Supplementary Materials

The following supporting information can be downloaded at: https://www.mdpi.com/article/10.3390/nu18142294/s1, Supplementary Data S1. HPLC-based chemo-profile of RGSF and ginsenoside components; Supplementary Data S2: NASH-CRN (nonalcoholic steatohepatitis clinical research network) histopathological grading; Supplementary Data S3: qRT-PCR primer list.
