# Supplementary material for: Ginsenoside Rb1-Enriched Saponin Fraction Inhibits M1 Macrophage Polarization by Suppression of TLR4 Trafficking in Metabolic Dysfunction-Associated Alcoholic Liver Disease
Source: Nutrients. 2026 Jul 13;18(14):2294. doi: 10.3390/nu18142294 (PMC13414620; doi:10.3390/nu18142294)
Supplement: Supplementary file 1 [file nutrients-18-02294-s001.zip › nutrients-4404731-supplementary.pdf]

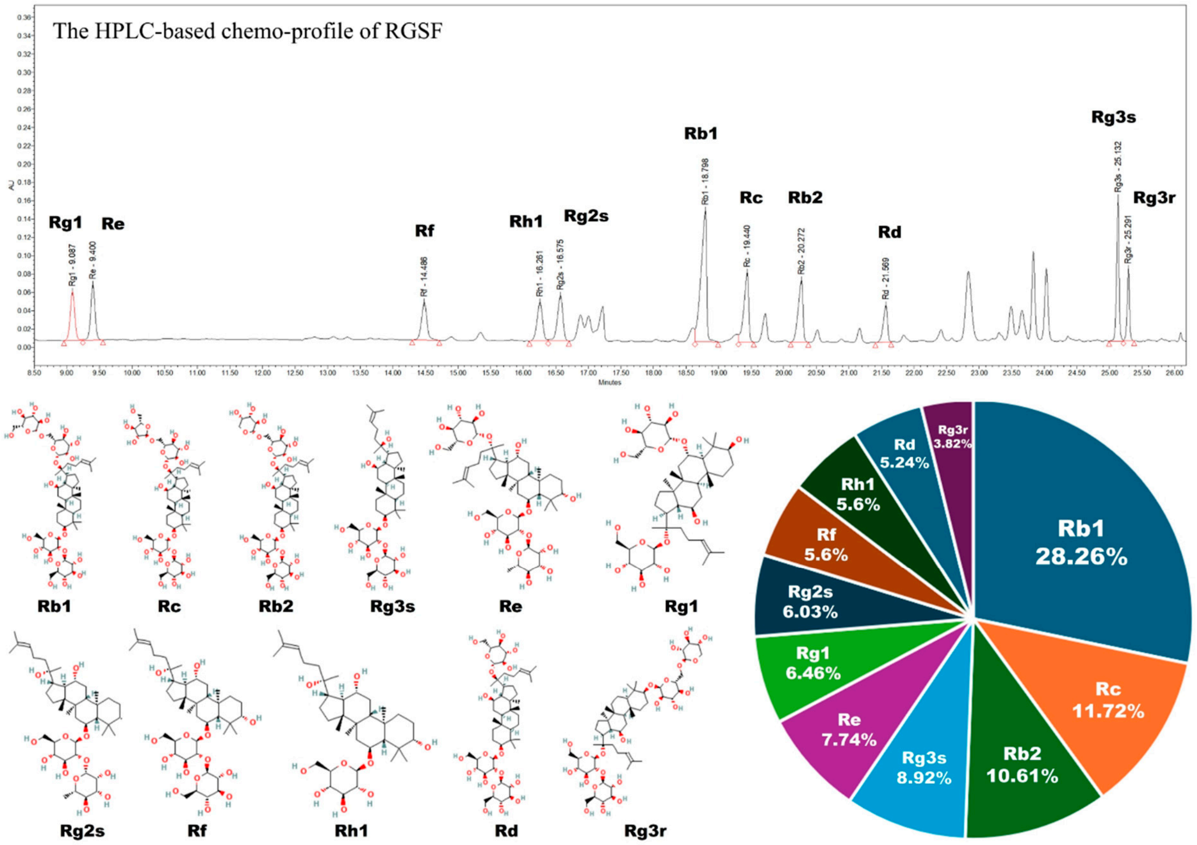

Supplementary Data S1. HPLC-based chemo-profile of RGSF and ginsenoside components.

HPLC: high performance liquid chromatography; RGSF: red ginseng saponin fraction.

Category	Score	Criteria
**Steatosis**	0	<5%
	0.5	6-15%
	1	16-25%
	1.5	26-33%
	2	34-50%
	2.5	51-66%
	3	67-75%
	4	76-100%
**Lobular inflammation**	0	No foci
	1	<2 foci per x200 field

	2	2-4 foci per x200 field
	3	>4 foci per x200 field

Supplementary Data S2. NASH-CRN (nonalcoholic steatohepatitis clinical research network) histopathological grading

Target gene	Primer sequences (5'→3')		Species
TNF-α	Forward	AGGCTGCCCCGACTACGT	mouse
	Reverse	GACTTTCTCCTGGTATGAGATAGCAAA	mouse
IL-1β	Forward	CTTTGAAGTTGACGGACCC	mouse
	Reverse	TGAGTGATACTGCCTGCCTG	mouse
iNOS	Forward	AATCTTGGAGCGAGTTGTGG	mouse
	Reverse	CAGGAAGTAGGTGAGGGCTTG	mouse
IL-10	Forward	GCCTTGCAGAAAAGAGAGCT	mouse
	Reverse	AAAGAAAGTCTTCACCTGGA	mouse
MRC1	Forward	CTCTGTTCAGCTATTGGACGC	mouse
	Reverse	CGGAATTTCTGGGATTCAGCTTC	mouse
Arg-1	Forward	CTCCAAGCCAAAGTCCTTAGAG	mouse
	Reverse	GGAGCTGTCATTAGGGACATC	mouse
18s	Forward	ACGGAAGGGCACCACCAGGA	mouse
	Reverse	CACCACCACCCACGGAATCG	mouse

Supplementary Data S3. qRT-PCR primer list.

qRT-PCR: quantitative real-time polymerase chain reaction; TNF-α: tumor necrosis factor alpha; IL-1β: interleukin 1 beta; iNOS: inducible nitric oxide synthase; IL-10: interleukin 10; MRC1: mannose receptor C-type 1; Arg-1: arginase 1.
